# Supplementary material for: NeutrobodyPlex—monitoring SARS‐CoV‐2 neutralizing immune responses using nanobodies
Source: EMBO Rep. 2021 Apr 27;22(5):e52325. doi: 10.15252/embr.202052325 (PMC8097376; doi:10.15252/embr.202052325)
Supplement: Supplementary file 1 — Appendix [file EMBR-22-e52325-s002.pdf]

Appendix:

NeutrobodyPlex - monitoring SARS-CoV-2 neutralizing immune responses using nanobodies

Teresa R. Wagner[1,2], Elena Ostertag[3]*; Philipp D. Kaiser[2], Marius Gramlich[2], Natalia Ruetalo[4], Daniel Junker[2], Julia Haering[2], Bjoern Traenkle[2], Matthias Becker[2], Alex Dulovic[2], Helen Schweizer[5], Stefan Nueske[5], Armin Scholz[5], Anne Zeck[2], Katja Schenke-Layland[2,6,7,8], Annika Nelde[6,9,10], Monika Strengert[12,13], Juliane S. Walz[6,9,10,11], Georg Zocher[3], Thilo Stehle[3,14], Michael Schindler[4], Nicole Schneiderhan-Marra[2] and Ulrich Rothbauer[1,2,6#]*

Addresses

[1] Pharmaceutical Biotechnology, Eberhard Karls University, Tuebingen, Germany

[2] Natural and Medical Sciences Institute at the University of Tuebingen, Reutlingen, Germany

[3] Interfaculty Institute of Biochemistry, Eberhard Karls University, Tuebingen, Germany

[4] Institute for Medical Virology and Epidemiology of Viral Diseases, University Hospital Tuebingen, Tuebingen, Germany

[5] Livestock Center of the Faculty of Veterinary Medicine, Ludwig Maximilians University, Oberschleissheim, Germany

[6] Cluster of Excellence iFIT (EXC2180) “Image-Guided and Functionally Instructed Tumor Therapies”, Eberhard Karls University, Tuebingen, Germany

[7] Department of Women’s Health, Research Institute for Women’s Health, Eberhard Karls University, Tuebingen, Germany

[8] Department of Medicine/Cardiology, Cardiovascular Research Laboratories, David Geffen School of Medicine at UCLA, Los Angeles, CA, USA

[9] Clinical Collaboration Unit Translational Immunology, German Cancer Consortium (DKTK), Department of Internal Medicine, University Hospital Tuebingen, Tuebingen, Germany

[10] Institute for Cell Biology, Department of Immunology, Eberhard Karls University, Tuebingen, Germany

[11] Dr. Margarete Fischer-Bosch Institute of Clinical Pharmacology and Robert Bosch Center for Tumor Disease, RBCT, Stuttgart, Germany

[12] Department of Epidemiology, Helmholtz Centre for Infection Research, Braunschweig, Germany

[13] TWINCORE GmbH, Centre for Experimental and Clinical Infection Research, a joint venture of the Hannover Medical School and the Helmholtz Centre for Infection Research, Hannover, Germany

[14] Vanderbilt University School of Medicine, Nashville, TN, USA

* both authors contributed equally to this work

# corresponding author

Correspondence:

Prof. Dr. Ulrich Rothbauer, Natural and Medical Sciences Institute at the University of Tuebingen

Markwiesenstr. 55, 72770 Reutlingen, Germany.

E-mail: ulrich.rothbauer@uni-tuebingen.de

Phone: +49 7121 51530-415

Fax: +49 7121 51530-816

Orcid ID: 0000-0001-5923-8986

Table of Content

Appendix Figures 4

Appendix Figure S1 - Affinities of Nbs determined by biolayer interferometry. 4

Appendix Figure S2 - Selected Nbs neutralize SARS-CoV-2 infection. 5

Appendix Figure S3 - Epitope binning of Nbs by biolayer interferometry. 6

Appendix Figure S4 - Differential HDX and sequence coverage of RBD upon Nb binding. 9

Appendix Figure S5 - NM1230 binding onto a spike in 'up/down/down' conformation and mapping of RBD:NM1230 interaction sites. 10

Appendix Figure S6- Influence of RBD mutations on NM1226 and NM1230 binding 12

Appendix Figure S7 – Affinities of Nbs against mutated versions of RBD derived from B.1.1.7 and B.1.351. 13

Appendix Figure S8 – Biparatopic NM1267 displaces neutralizing IgG binding the RBD:ACE2 interaction site. 14

Appendix Tables 15

Appendix Table S1 – Amino acid sequences of RBD-specific Nbs. 15

Appendix Table S2 – Data collection and refinement statistics for the RBD:NM1226 and RBD:NM1230 complex structures. 16

Appendix Table S3 - NeutrobodyPlex data illustrated in Fig 5B, C. 17

Appendix Table S4 - Data of control samples (C1-C4) from healthy donors tested in the NeutrobodyPlex and viral infection assay. 19

References 20

Appendix Figures

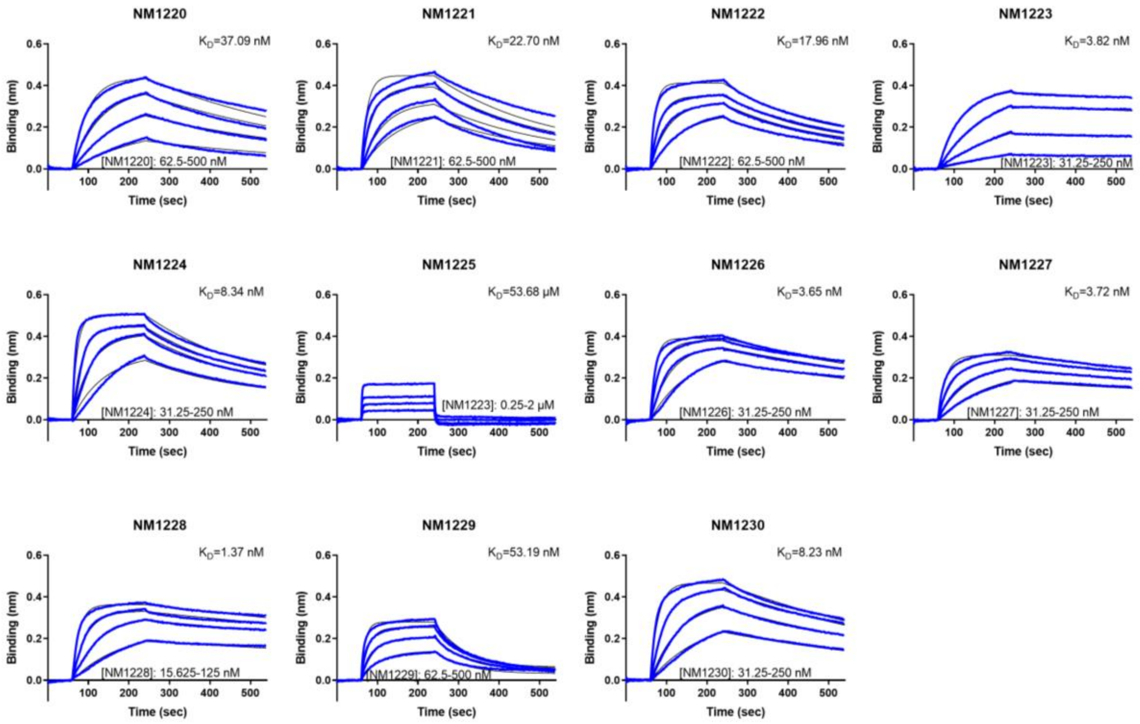

Appendix Figure S1 - Affinities of Nbs determined by biolayer interferometry.

Sensograms of BLI-based affinity measurements of 11 identified RBD-specific Nbs are shown. Biotinylated RBD was immobilized on streptavidin biosensors and kinetic measurements were performed by using four concentrations of purified Nbs ranging from 15.6 nM - 2 μM.

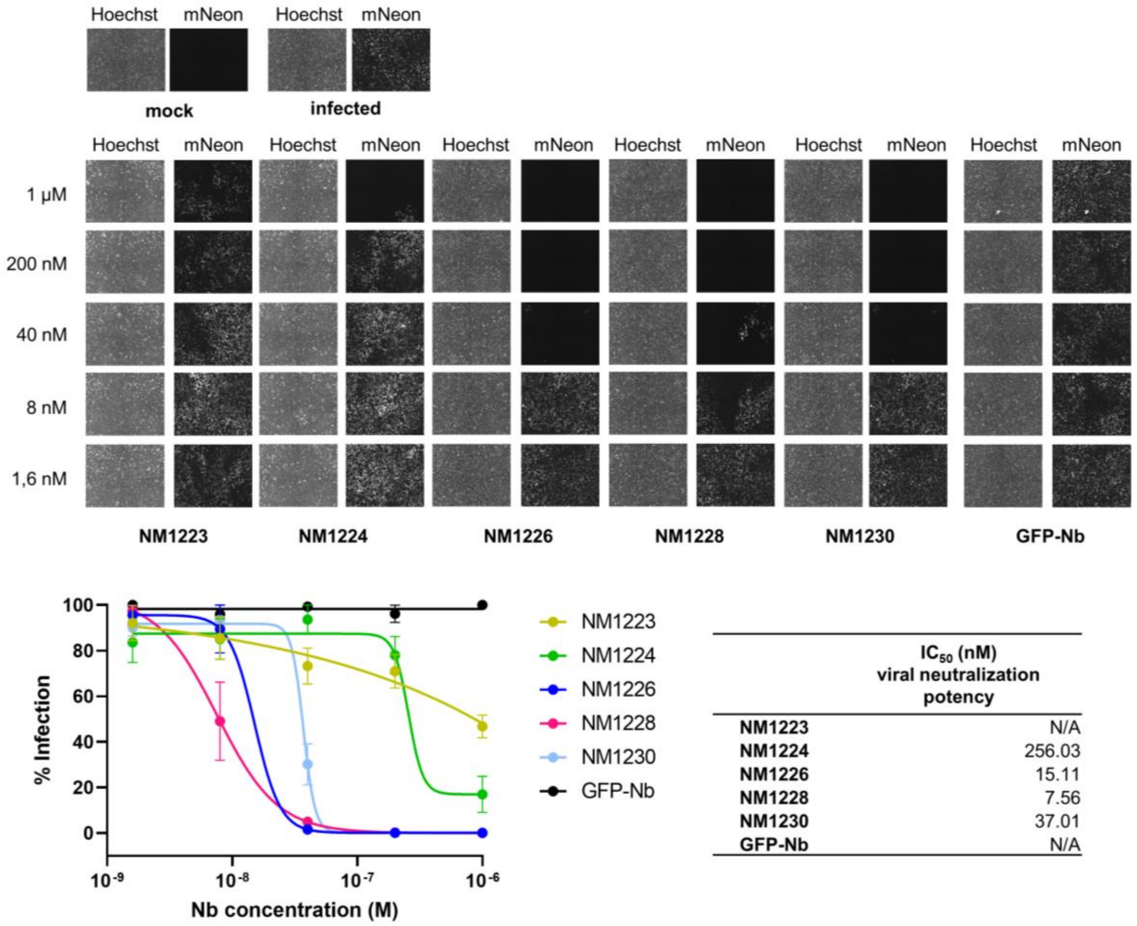

Appendix Figure S2 - Selected Nbs neutralize SARS-CoV-2 infection.

Neutralization potency of NM1223, NM1224, NM1226, NM1228 and NM1230 was analyzed in Caco-2 cells using the SARS-CoV-2-mNG strain. As negative control, the GFP-Nb was used. Representative images of human Caco-2 cells upon infection with SARS-CoV-2 expressing mNeonGreen either in presence or absence of serial dilutions of RBD Nbs are shown. Infection rate normalized to virus-only infection control is illustrated as percent of infection (% Infection). IC_{50} value was calculated from a four-parametric sigmoidal model and data are presented as mean ± s.e.m. of three biological replicates (n = 3).

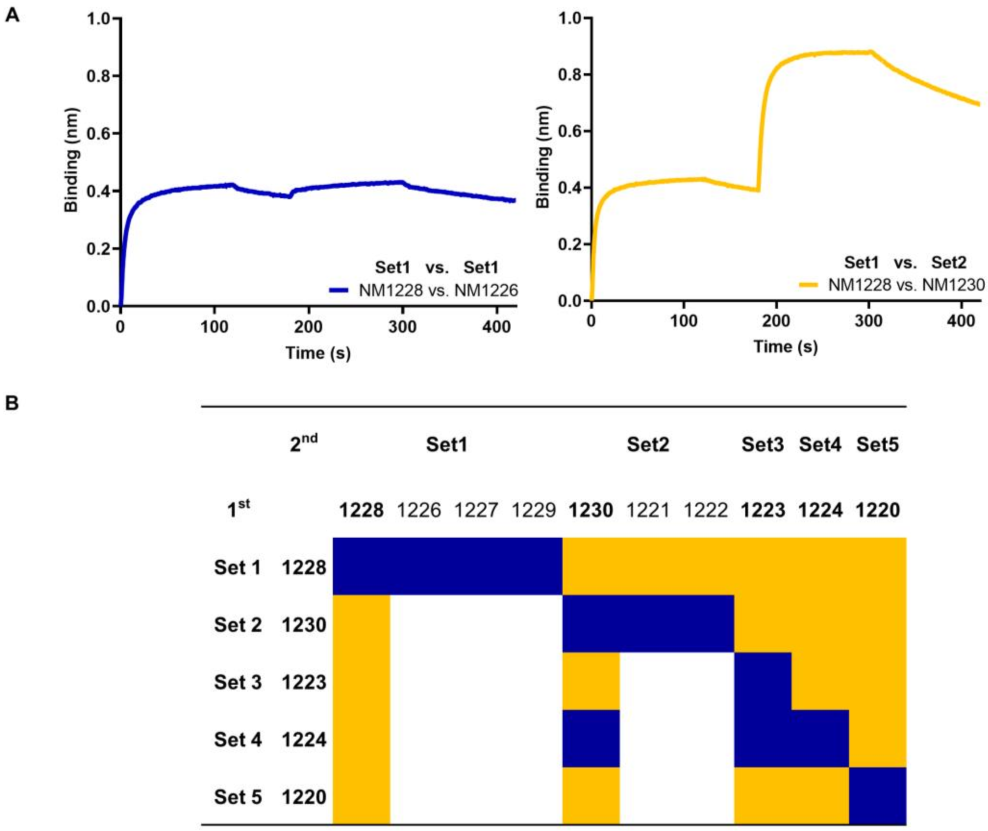

Appendix Figure S3 - Epitope binning of Nbs by biolayer interferometry.

A Representative sensograms of single measurements of Nbs affiliated to the same Nb-Set (NM1228/ NM1226, blue) and two different Nb-Sets (NM1228/ NM1230, orange) are shown.

B Heat map illustration of competitive Nb epitope binning on RBD using BLI. Rows and columns represent the loading of the first and second Nb, respectively. Blue colored squares illustrate no additional binding of the second Nb meaning both Nbs belong to the same Nb-Set. Orange colored squares represent additional binding of the second Nb, hence these Nbs belong to different Nb-Sets.

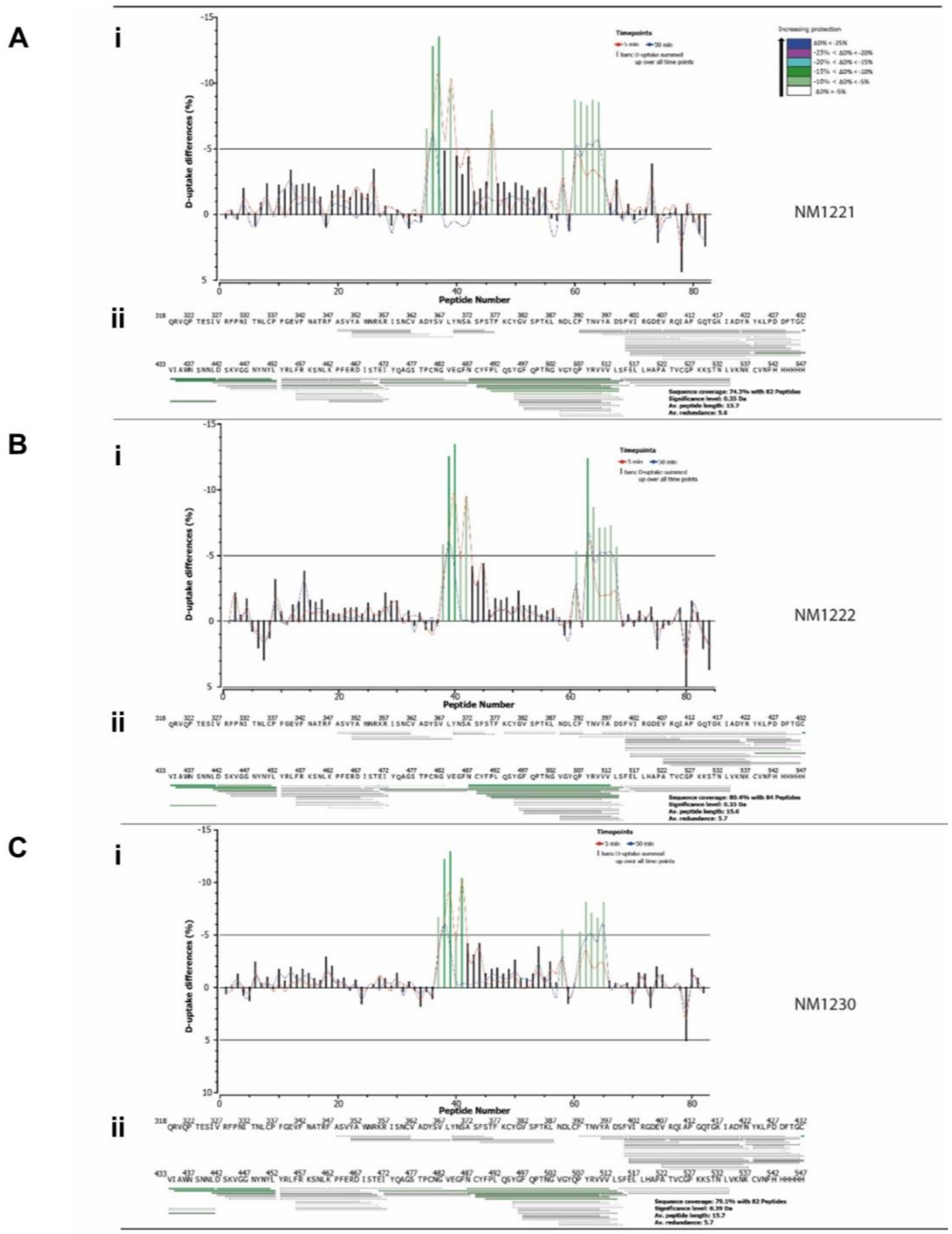

A
i
ii
NM1221
B
NM1222
C
NM1230
D-uptake differences (%)
Peptide Number
Timepoints
Increasing protection

104

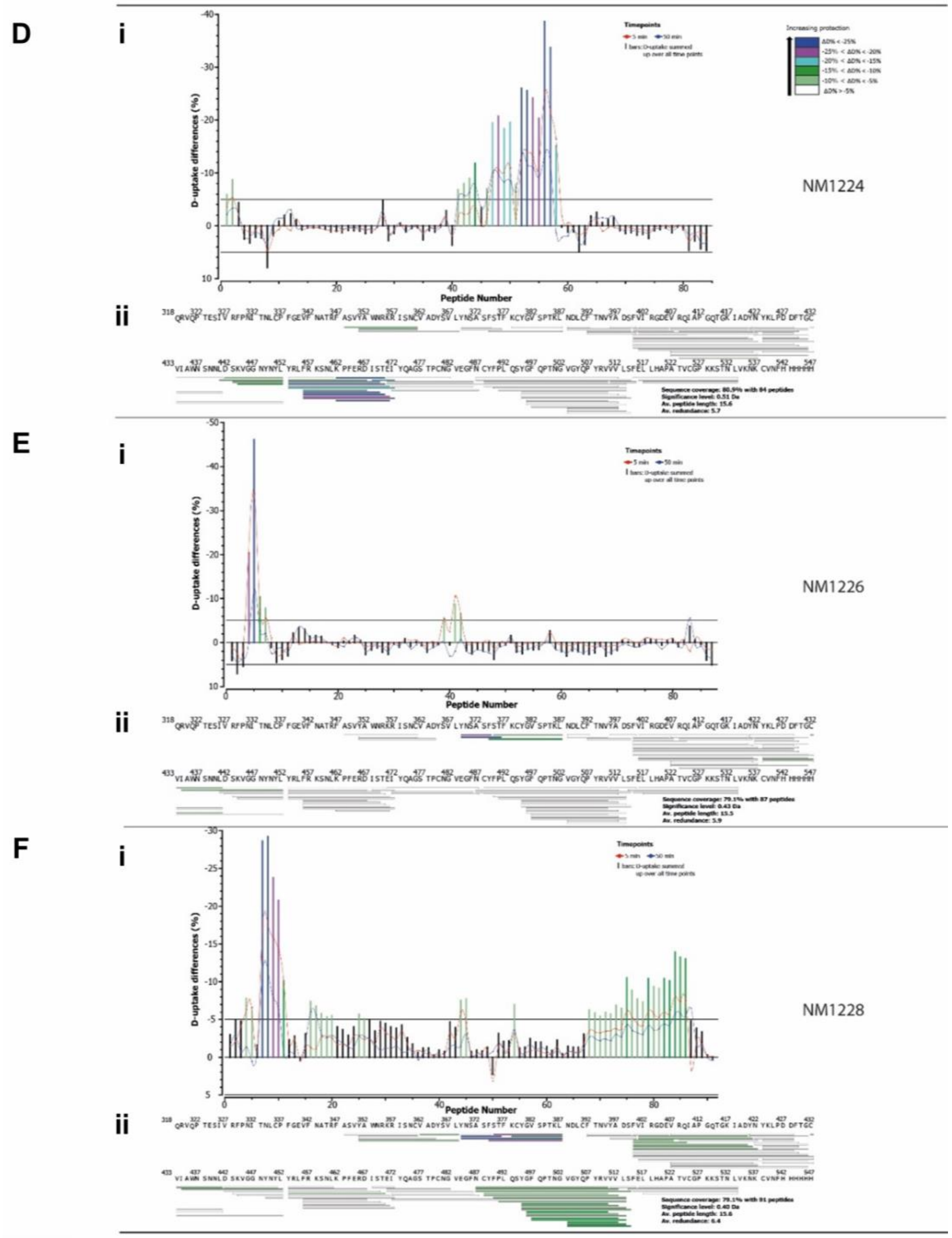
D
i
ii
E
i
ii
F
i
ii
D-uptake differences (%)
Peptide Number
Timepoints
NM1224
NM1226
NM1228

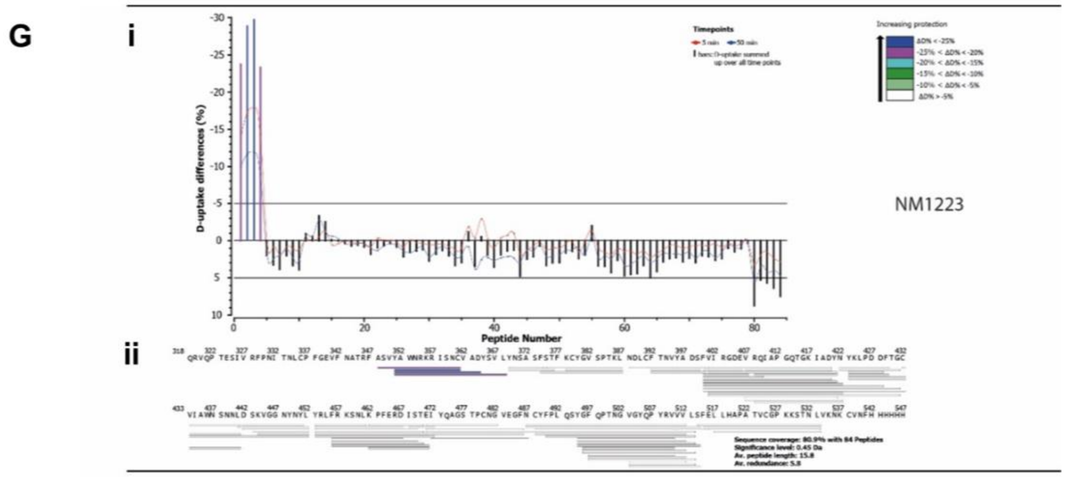

Appendix Figure S4 - Differential HDX and sequence coverage of RBD upon Nb binding.

A NM1221

B NM1222

C NM1230

D NM1224

E NM1226

F NM1228

G NM1223

i Residual differential HDX of partially overlapping peptic peptides numbered from the N- to the C-terminus and from short to long peptides. The minimum significant deuterium uptake difference (significance level) was calculated by HDExaminer from the variance of triplicate runs. On this basis, a minimum threshold of 3% deuterium difference was used to define regions as unprotected upon Nb binding. Peptides showing higher protection than 5% were considered as protected and corresponding bars were color-coded according to the legend.

ii Sequence coverage map of peptides validated for HDX data analysis for each nanobody-RBD pair. Peptides above the 5% protection threshold were color-coded using the colors from (i).

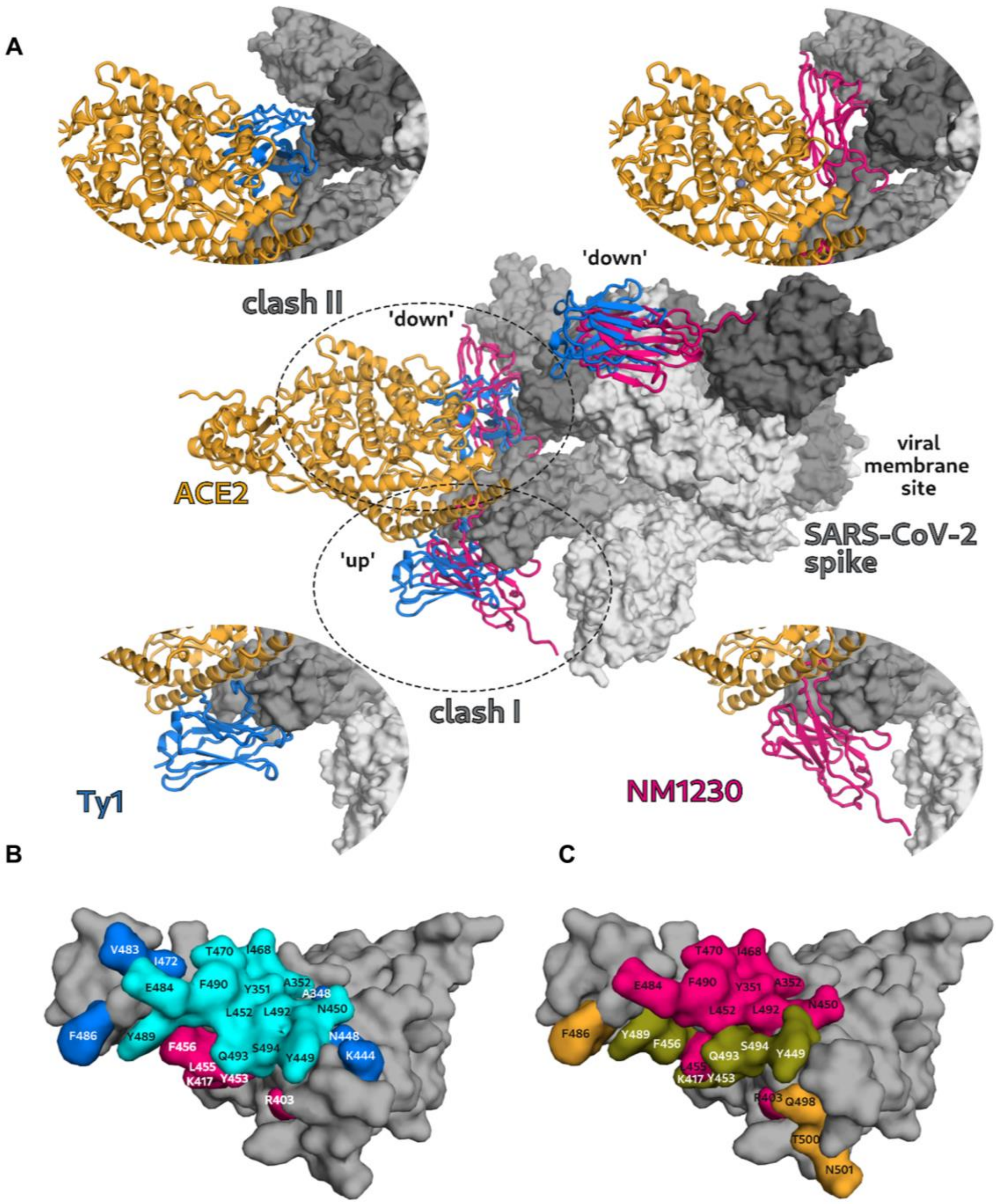

Appendix Figure S5 - NM1230 binding onto a spike in 'up/down/down' conformation and mapping of RBD:NM1230 interaction sites.

A Alignment of the SARS-CoV-2 spike:Ty1-Nb (blue) cryoEM structure (pdb code: 6ZXN) (Hanke *et al*, 2020) with the RBD:NM1230 complex. The ACE2 (orange) (pdb code: 6M17) and the NM1230 (pink) and Ty1-Nb (blue) are depicted as cartoons, whereas the spike trimer is shown in surface representation (grey). The neutralizing effect of NM1230 and Ty1-Nb is shown by expected collisions (clash I and clash II) with ACE2 in the alignment. Additional

close-up views of the individual Nb positions are shown to highlight the differences in Nb binding and ACE2 blocking.

B Individual binding sites of NM1230 (pink) and the Ty1-Nb (blue) (Hanke *et al.*, 2020) as well as common interaction residues (cyan) are highlighted on the surface representation of RBD.

C Comparison of the ACE2 interaction site and NM1230 epitope on RBD. Common residues are shown in olive, whereas residues exclusively in contact with ACE2 and NM1230 are colored in orange and pink, respectively. All interactions of NM1230, Ty1-Nb and ACE2 are depicted using a distance cut-off of < 4 Å to the surface of RBD.

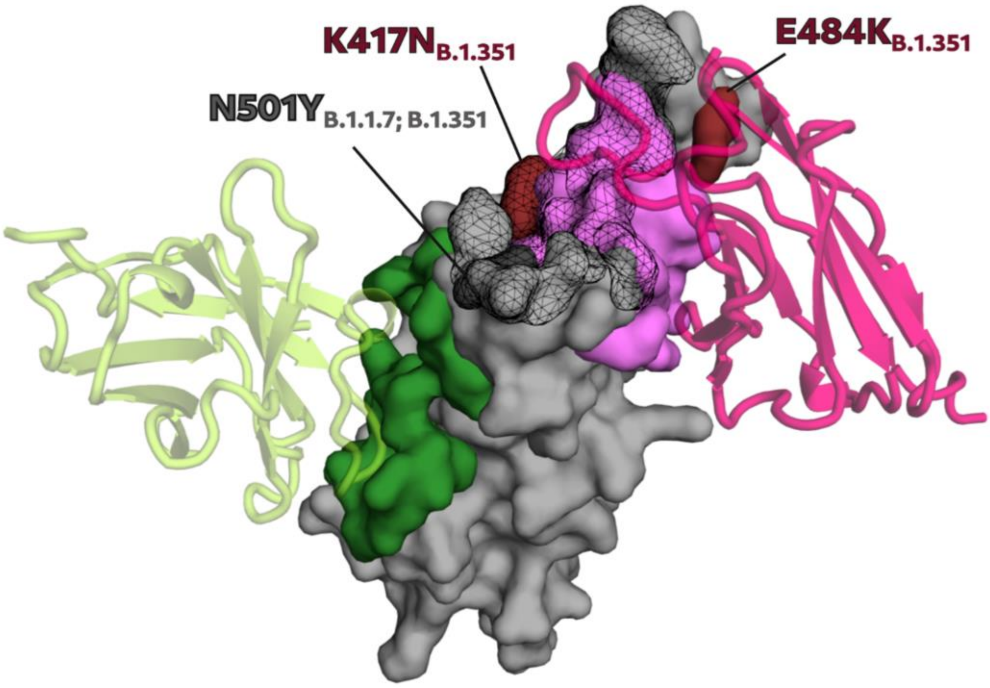

Virus Strain	RBD Mutation
**B.1.1.7 (UK)**	N501Y
**B.1.351 (South Africa)**	N501Y, E484K, K417N

Appendix Figure S6- Influence of RBD mutations on NM1226 and NM1230 binding.

NM1226 (light green) and NM1230 (magenta) are shown as cartoon with their corresponding binding epitopes on the RBD (surface representation) surface in dark green and light pink, respectively. Mutations on the RBD and in the binding epitope of NM1230, discovered in B.1.1.7 (United Kingdom) and B.1.351 (South Africa) SARS-CoV-2 strains, are highlighted in dark grey and dark red, respectively. In addition, the ACE2 interaction site on RBD is indicated as black mesh.

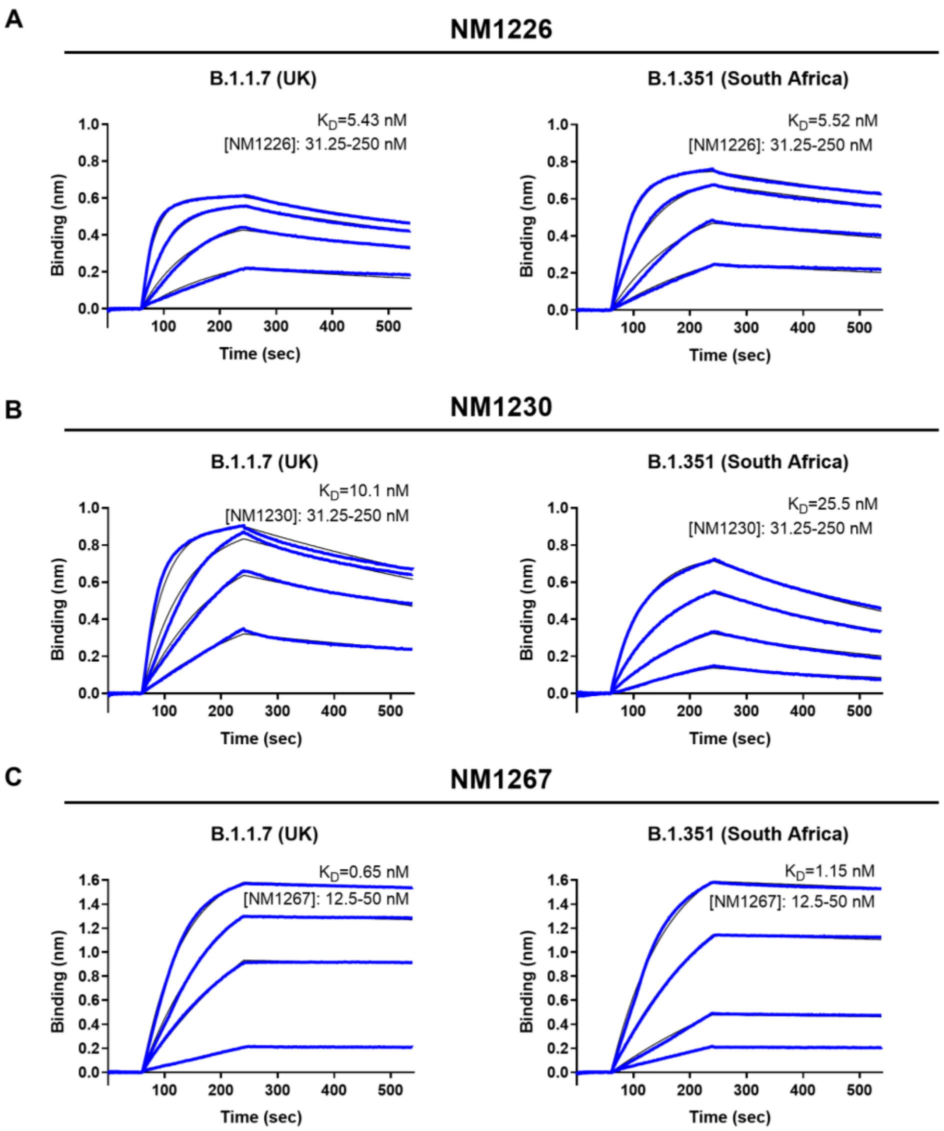

Appendix Figure S7 – Affinities of Nbs against mutated versions of RBD derived from B.1.1.7 and B.1.351.

Sensograms of BLI-based affinity measurements of selected RBD-specific Nbs are shown. Biotinylated RBD mutants derived from B.1.1.7 (UK) or B.1.351 (South Africa) were immobilized on streptavidin biosensors and kinetic measurements were performed by using four concentrations of purified Nbs ranging from 12.5 nM – 250 nM.

A NM1226

B NM1230

C NM1267

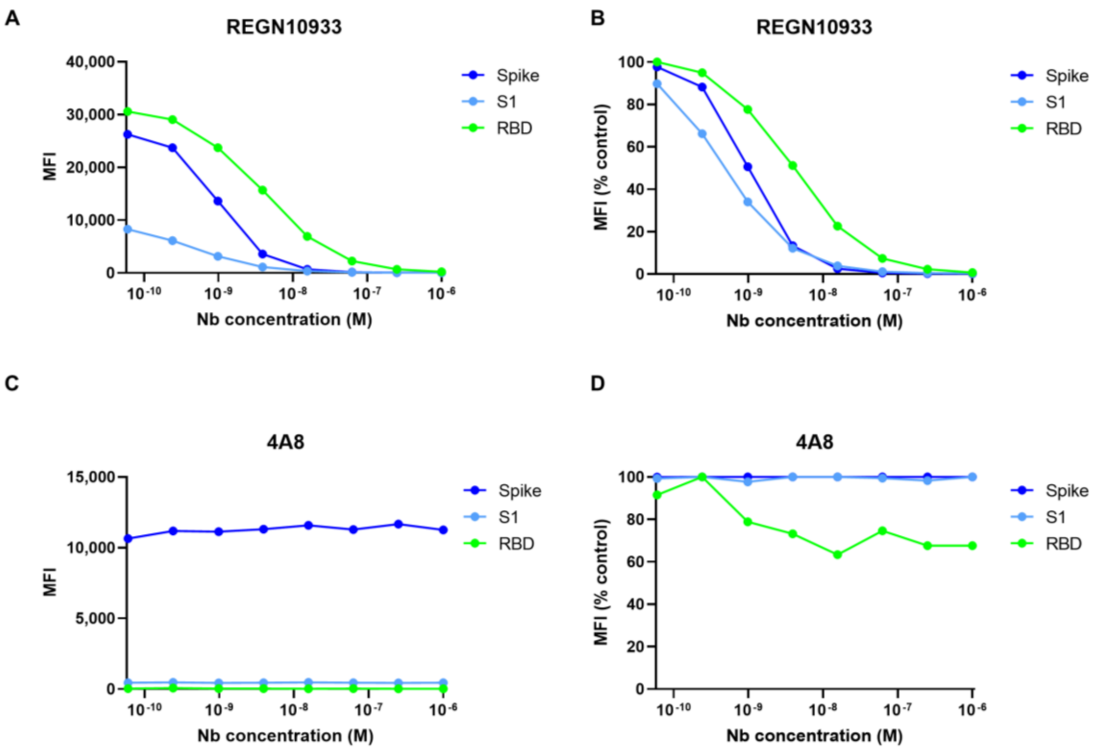

Appendix Figure S8 – Biparatopic NM1267 displaces neutralizing IgG binding the RBD:ACE2 interaction site.

Antigen-coated beads comprising RBD, S1 or spike were co-incubated with purified IgGs and a dilution series of NM1267 (1 µM to 6 pM). Mean fluorescent intensities (MFI) derived from antigen-bound IgGs in the presence of bipNb and MFIs normalized to the MFI values of IgG-only samples ((MFI (% control)) are illustrated.

A, B Anti-Spike RBD IgG clone REGN10933 (Hansen *et al*, 2020).

C, D Anti-Spike NTD IgG clone 4A8 (Chi *et al*, 2020)

Appendix Tables

Appendix Table S1 – Amino acid sequences of RBD-specific Nbs.

RBD-specific Nb	Amino Acid Sequence
**NM1220**	QVQLVESGGGLVQPGESLRLSCVAYGNMLRGYVVGWYRQAPGKQRELVAGIDTSGEKKKYADA<br>VKGRFTISRDNAGNTVYLQMNSLKPEDTAVYYCNADAPWPPRPYSVIGTRTGYWGRGSPVTVSS
**NM1221**	DVQLVESGGGLVQPGGSLTLSCVGSGFLFSGYAMNWYRQAPGKALELVAGISNAGDITHYEEAM<br>KGRVAISRVNDKNTVYLQMDDLKPEDTAVYRCHAPGVRVASGERNDVWGQGTQVTVSS
**NM1222**	EVQLVESGGGLVRPGGSLRLSCVGSGFTFSGYAINWYRQAPGKALELVAGISNAGDLTHYEEAM<br>KGRVAISRANDKNTVYLQMDDLKPEDTAVYRCHAPGVRVGTGERNDVWGQGAQVTVSS
**NM1223**	EVQLVESGGGLVQPGGSLRLSCSASGFAFSSVSMSWVRLLPGKGTEWVAEIDRDGGNGNYEDS<br>VKGRFTISRDNAKNTLFLQMNSLVPEDTALYYCRLGTRDHIMSGWGPGAPVTVSS
**NM1224**	DVQLVESGGSLVQPGGSLRLSCETSRSSLDAYAIGWFRQAPGKEREGVASISSSSMRTEYADSV<br>KGRFTISRDNAKNTAYLDMNSLKPEDTAVYYCAAAGEYGRAWPGLDWYEFEYEGPGSPVTVSS
**NM1225**	QVQLVESGGGLVQPGGSLRLSCAASGNILRVHDMGWYRQAPGKQREYVAMITHGGITNYIDSVK<br>GRFTISRDNAKNTVYLQMNSLKPEDTAVYYCHAVLSSALNGVTETSSNWGQGTQVTVSS
**NM1226**	QVQLVESGGGSVQPGGSLRLSCLGSGSLDYYAIGWFRQAPGKEREGVSCIASSGDRTIYADSVK<br>GRFTISRDYGKNTVYLQMNSLKPEDTAMYYCAALQGSYYYTGFVANEYDYWGQGAPVTVSS
**NM1227**	EVQLVESGGGLVQPGGSLTLSCETSGRHFDIDDMGWYRQAPGKQRELVACITTESSTTYADAVK<br>GRFTISRDNPDNTVYLQMTNLKPEDTAVYYCNAEMHPRSLDYALGNRDYWGQGAPVTVSS
**NM1228**	DVQLVESGGGLVRPGGSLRLSCESSGRHFDIDTMGWYRQAPGKQRELVASITSEKSTVYADALK<br>GRFTISRDIPDNNVYLQMNNLKPEDTAVYYCNAKMDPHSLDYALGNQVFWGQGSLVTVSS
**NM1229**	EVQLQESGGGLVQPGESLRLSCAASGRTHDWYTMGWFRQAPGKEREFVARINWSSGMTYYAD<br>SVKGRFTISRDNPKNTVYLQMNSLTPDDTAVYYCNVHPFTSPDYWGQGTRVTVSS
**NM1230**	QVQLVESGGGLVRPGGSLRLSCVGSGFTFSGYAMNWYRQAPGKALELVAGISNAGDLTHYEEP<br>MKGRVAISRANDKNTVYLQMDDLKPEDTAVYRCHAPGVRVGTGERKDVWGQGAQVTVSS

Appendix Table S2 – Data collection and refinement statistics for the RBD:NM1226 and RBD:NM1230 complex structures.

Values in parentheses are for the highest resolution shell.

	RBD:NM1226 complex (PDB: 7NKT)	RBD:NM1230 complex (PDB: 7B27)
**Resolution (Å)**	46.1-2.3 (2.44-2.30)	46.5-2.90 (2.98-2.90)
**Space group**	$I4_1$	$P4_32_12$
**Unit cell**		
**a, c (Å)**	128.09, 77.68	63.29, 411.91
**No of reflections**	255015 (38715)	112109 (8061)
**unique reflections**	28115 (4500)	18493 (1340)
**Redundancy**	9.07	6.06
**Completeness (%)**	99.9 (99.6)	93.5 (95.7)
**I/σ (I)**	19.5 (1.42)	7.8 (1.15)
**CC1/2**	99.9 (48.4)	99.8 (66.2)
**Wilson B (Å^2)**	57	61
**R_{meas} (%)**	9.5 (156.8)	20.7 (186.2)
**R_{work} / R_{free} (%)**	18.4 / 22.4	26.6 / 30.5
**Rmsd**		
**Bond angle (°)**	1.45	1.21
**Bond length (Å)**	0.011	0.016
**Average B-Factor (Å^2)**		
**RBD (chain A/B)**	61.7	74.6 / 75.2
**NM1230 (chain C/D)**		64.5 / 81.2
**NM1226(chain B)**	70.7	
**water**	55.5	
**Ramachandran**		
**Favoured (%)**	97.0	86.3
**Outlier (%)**	0.3	3.9

Appendix Table S3 - NeutrobodyPlex data illustrated in Fig 5B, C.

Values as MFI and MFI (% control).

	**# 225**						
	Conc. NM1267 [nM]	MFI S1	MFI S1 (% control)	MFI Spike	MFI Spike (% control)	MFI RBD	MFI RBD (% control)
NM1267	1000.00	4453	49.58	26793	69.78	103	0.49
	250.00	4425	49.27	26898	70.05	162	0.77
	62.50	4377	48.74	26219	68.28	336	1.60
	15.63	4423	49.24	26126	68.04	1043	4.95
	3.91	4769	53.10	27975	72.85	3717	17.66
	0.98	5304	59.06	28713	74.78	9823	46.66
	0.24	6556	73.00	34834	90.72	16580	78.76
	0.06	8069	89.84	37128	96.69	19723	93.69
Control n=2 (Serum 1:400)	-	9076		38978		21104	
	-	8886		37819		20999	
	**# 289**						
	Conc. NM1267 [nM]	MFI S1	MFI S1 (% control)	MFI Spike	MFI Spike (% control)	MFI RBD	MFI RBD (% control)
NM1267	1000.00	503.0	15.31	15943	69.07	258	2.29
	250.00	512.0	15.58	16250	70.40	291	2.59
	62.50	527.5	16.05	16316	70.69	349	3.10
	15.63	547.0	16.65	16319	70.70	639	5.68
	3.91	641.0	19.51	16263	70.46	1667	14.82
	0.98	908.0	27.63	17369	75.25	4753	42.26
	0.24	1630.5	49.62	21303	92.29	8985	79.89
	0.06	2713.0	82.56	23808	103.15	11377	100.00
Control n=2 (Serum 1:400)	-	3320.0		23012		11250	
	-	3252.5		23152		11244	
	**# 265**						
	Conc. NM1267 [nM]	MFI S1	MFI S1 (% control)	MFI Spike	MFI Spike (% control)	MFI RBD	MFI RBD (% control)
NM1267	1000.00	1217	47.00	14574	73.63	93	1.36
	250.00	1264	48.81	15273	77.16	118	1.74
	62.50	1230	47.50	14896	75.26	174	2.56
	15.63	1255	48.48	14788	74.71	351	5.18
	3.91	1310	50.61	14993	75.75	947	13.96
	0.98	1549	59.84	15985	80.76	2535	37.38
	0.24	1881	72.65	17932	90.60	4648	68.52
	0.06	2225	85.96	19524	98.64	6147	90.63
Control n=2 (Serum 1:400)	-	2567		19781		6666	
	-	2610		19805		6899	

				# 272			
	Conc. NM1267 [nM]	MFI S1	MFI S1 (% control)	MFI Spike	MFI Spike (% control)	MFI RBD	MFI RBD (% control)
NM1267	1000.00	159	25.37	15289	86.71	48	2.05
	250.00	163	25.93	14686	83.29	53	2.24
	62.50	153	24.41	14857	84.26	65	2.77
	15.63	163	26.01	14923	84.63	116	4.95
	3.91	192	30.63	14895	84.47	321	13.70
	0.98	237	37.73	15036	85.27	853	36.40
	0.24	335	53.45	16833	95.47	1647	70.28
	0.06	531	84.72	17305	98.14	2165	92.38
Control n=2 (Serum 1:400)	-	611		17575		2329	
	-	643		17690		2359	

				# 159			
	Conc. NM1267 [nM]	MFI S1	MFI S1 (% control)	MFI Spike	MFI Spike (% control)	MFI RBD	MFI RBD (% control)
NM1267	1000.00	96	49.61	12792	99.38	37	5.07
	250.00	87	45.19	11708	90.96	37	5.00
	62.50	90	46.75	11503	89.36	43	5.83
	15.63	79	41.04	11340	88.10	51	6.99
	3.91	82	42.60	11263	87.50	92	12.54
	0.98	86	44.68	11437	88.85	257	35.23
	0.24	117	60.78	12483	96.98	531	72.79
	0.06	166	85.97	13044	100.00	681	93.28
Control n=2 (Serum 1:400)	-	191		13077		758	
	-	194		12668		702	

Appendix Table S4 - Data of control samples (C1-C4) from healthy donors tested in the NeutrobodyPlex and viral infection assay.

NeutrobodyPlex								
	C1		C2		C3		C4	
	MFI RBD	MFI RBD (% control)	MFI RBD	MFI RBD (% control)	MFI RBD	MFI RBD (% control)	MFI RBD	MFI RBD (% control)
1 µM NM1267	83	80.58	71	89.87	73	72.28	49	76.56
1 nM NM1267	103	100.00	103	100.00	89	88.12	80	100.00
Serum only	103	100.00	79	100.00	101	100.00	64	100.00

Viral Infection Assay				
**Serum Dilution**	**% Infection**			
	**C1**	**C2**	**C3**	**C4**
1:40	100.00	100.00	55.56	91.54
1:80	98.24	100.00	95.44	94.69
1:160	100.00	100.00	100.00	98.45
1:320	100.00	100.00	100.00	96.90
1:640	84.62	100.00	100.00	100.67
1:1280	95.77	91.11	99.26	70.12
1:2560	93.01	75.81	95.86	85.80
1:5120	83.85	94.74	94.39	100.00

References

Chi X, Yan R, Zhang J, Zhang G, Zhang Y, Hao M, Zhang Z, Fan P, Dong Y, Yang Y *et al* (2020) A neutralizing human antibody binds to the N-terminal domain of the Spike protein of SARS-CoV-2. *Science* 369: 650-655

Hanke L, Vidakovics Perez L, Sheward DJ, Das H, Schulte T, Moliner-Morro A, Corcoran M, Achour A, Karlsson Hedestam GB, Hallberg BM *et al* (2020) An alpaca nanobody neutralizes SARS-CoV-2 by blocking receptor interaction. *Nat Commun* 11: 4420

Hansen J, Baum A, Pascal KE, Russo V, Giordano S, Wloga E, Fulton BO, Yan Y, Koon K, Patel K *et al* (2020) Studies in humanized mice and convalescent humans yield a SARS-CoV-2 antibody cocktail. *Science* 369: 1010-1014
