# Supplementary material for: NeutrobodyPlex—monitoring SARS‐CoV‐2 neutralizing immune responses using nanobodies
Source: EMBO Rep. 2021 Apr 27;22(5):e52325. doi: 10.15252/embr.202052325 (PMC8097376; doi:10.15252/embr.202052325)
Supplement: Supplementary file 2 — Expanded View Figures PDF [file EMBR-22-e52325-s003.pdf]

Expanded View Figures

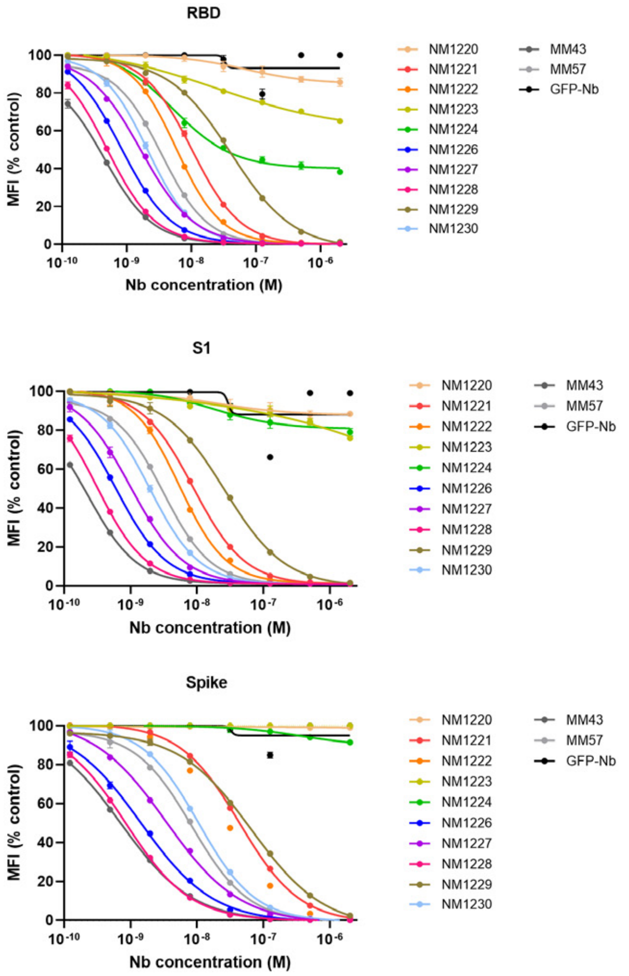

	IC_{50} (nM) ACE2:RBD Inhibition	IC_{50} (nM) ACE2:S1 Inhibition	IC_{50} (nM) ACE2:Spike Inhibition
**NM1220**	NA	NA	NA
**NM1221**	10.14	43.28	8.95
**NM1222**	5.80	27.28	5.50
**NM1223**	NA	NA	NA
**NM1224**	36.58	NA	NA
**NM1226**	0.82	1.44	0.63
**NM1227**	1.65	3.51	1.06
**NM1228**	0.50	0.85	0.32
**NM1229**	37.83	56.07	24.95
**NM1230**	2.12	10.57	1.96
**GFP-Nb**	NA	NA	NA
**MM43**	0.38	0.71	0.20
**MM57**	3.22	7.83	2.93

Figure EV1. ACE2-competing Nbs identified by multiplex binding assay.

Results from multiplex ACE2 competition assay are shown for the three spike-derived antigens: RBD, S1-domain (S1) and homotrimeric spike (Spike). Nbs were diluted from 2.1 µM to 0.12 nM in the presence ACE2 and antigen-bound ACE2 was measured. MFI signals were normalized to the maximum detectable signal per antigen given by the ACE2-only control. IC_{50} values were calculated from a four-parametric sigmoidal model. Data are presented as mean ± s.d. of three technical replicates.

Figure EV2. Epitope mapping of Nbs by HDX mass spectrometry.

Surface structure model of RBD showing the ACE2 interface and the HDX-MS epitope mapping results of RBD epitopes protected upon Nb binding are highlighted in different colors indicating the strength of protection. Accordingly, amino acid residues, which are part of the Nb recognized epitopes, are highlighted in the RBD sequence.

A Numbered amino acid residues of RBD (pdb code: 6M17 (Yan *et al*, 2020)) involved in the RBD:ACE2 interaction site (Lan *et al*, 2020; Yan *et al*, 2020) are shown in red.
B NM1226 (Nb-Set1)
C NM1228 (Nb-Set1)
D NM1230 (Nb-Set2)
E NM1221 (Nb-Set2)
F NM1222 (Nb-Set2)
G NM1224 (Nb-Set4)
H NM1223 (Nb-Set3)

▶

© 2021 The Authors

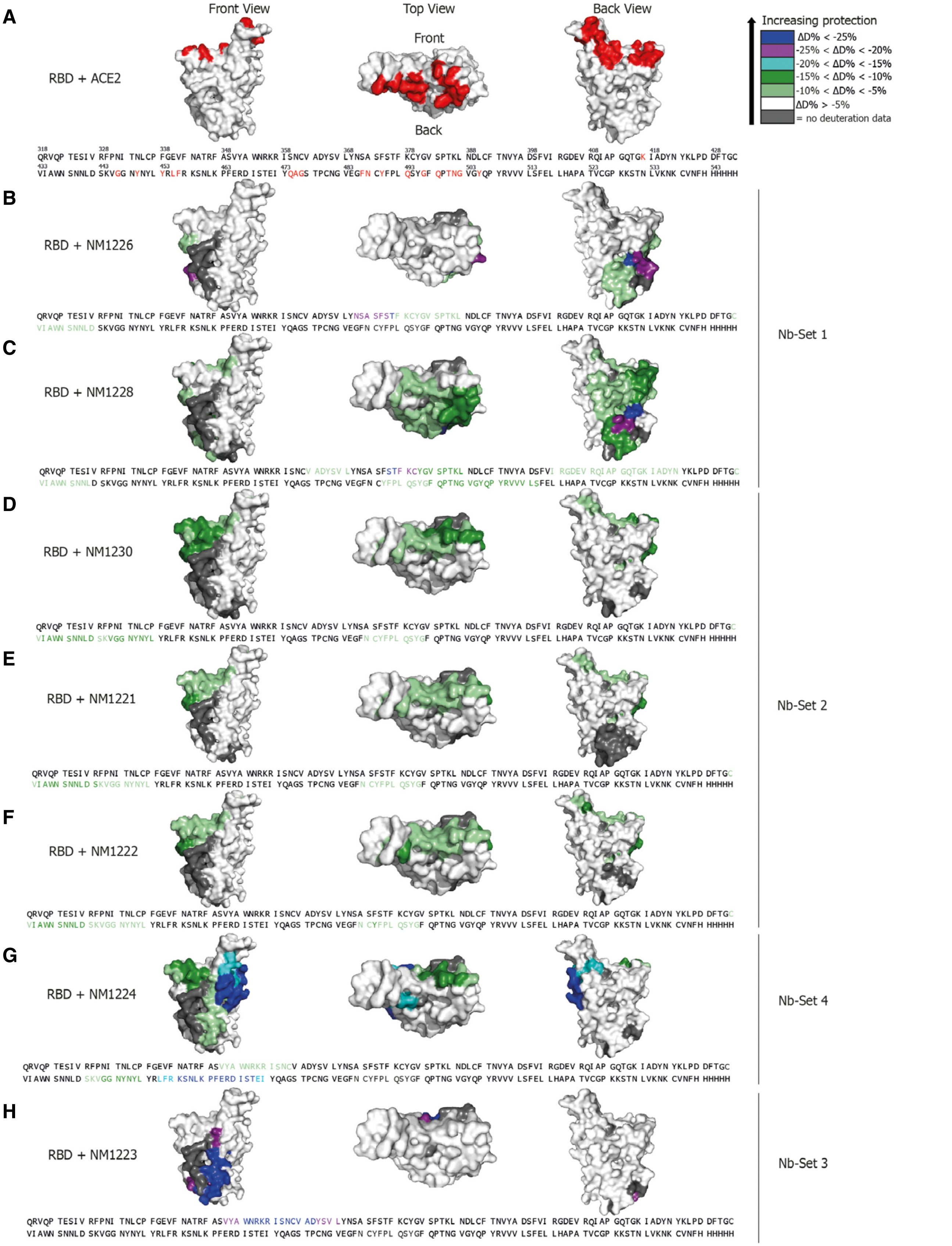

Figure EV2.

© 2021 The Authors

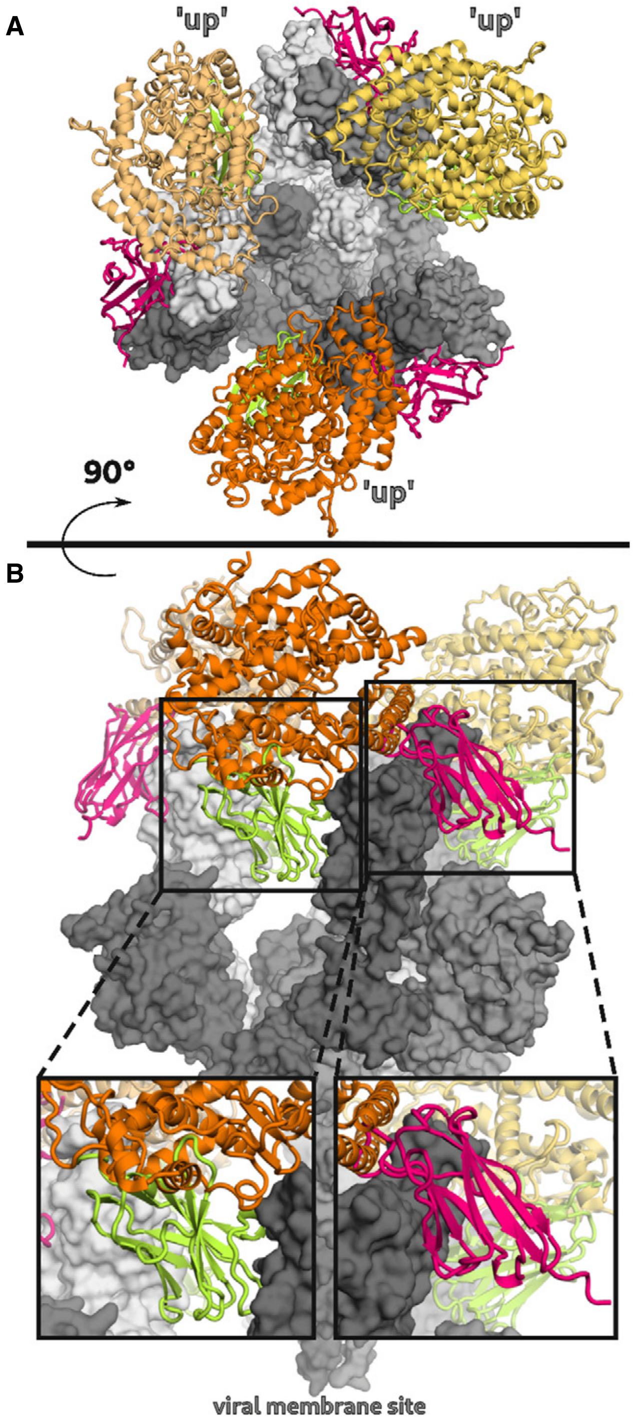

Figure EV3. Mechanism of ACE2 attachment inhibition by NM1226 and NM1230.

A, B (A) Top and (B) side view of the SARS-CoV-2 spike protein (gray) in complex with the ACE2 receptor (yellow, orange, dark orange) (pdb code: 7KMS) superposed on the RBD:Nb complexes NM1226 (light green) and NM1230 (magenta). A close-up view shown in black boxes elucidates how both Nbs block ACE2 receptor attachment.

© 2021 The Authors

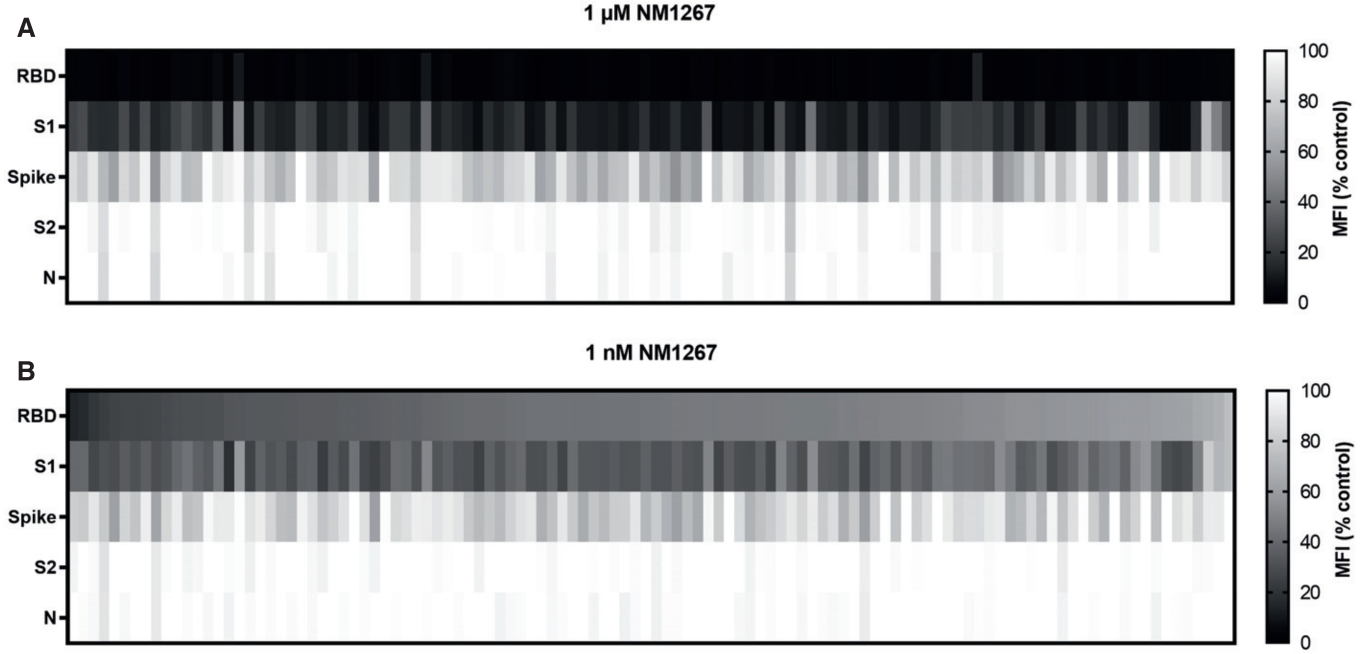

Figure EV4. NeutrobodyPlex enables a differentiated analysis on neutralizing IgGs.

Serum samples of 112 convalescent SARS-CoV-2-infected individuals were analyzed using the NeutrobodyPlex with antigen-coated beads comprising RBD, S1, spike, S2-domain (S2), and nucleocapsid (N) and two concentrations of NM1267 ($n = 1$). Light colored squares (high MFI (%control)) are indicative for IgGs outcompeting NM1267 from the RBD:ACE2 interface; dark colored squares (low MFI (% control)) show a continuous displacement of IgGs from serum samples in the presence of NM1267.

A NeutrobodyPlex NM1267 1 µM.
B NeutrobodyPlex NM1267 1 nM.

 © 2021 The Authors
